# DABS-MS: deep atlas-based segmentation using the Mumford–Shah functional

**DOI:** 10.1117/1.JMI.12.5.055002

**Published:** 2025-10-21

**Authors:** Hannah G. Mason, Jack H. Noble

**Affiliations:** a Vanderbilt University, Department of Computer Science, Nashville, Tennessee, United States; b Vanderbilt University, Department of Electrical and Computer Engineering, Nashville, Tennessee, United States

**Keywords:** atlas-based registration, deformation, Mumford–Shah functional, nonrigid deformation, segmentation, VoxelMorph, cochlear implants

## Abstract

**Purpose:**

Cochlear implants (CIs) are neural prosthetics used to treat patients with severe-to-profound hearing loss. Patient-specific modeling of CI stimulation of the auditory nerve fiber (ANF) can help audiologists improve the CI programming. These models require localization of the ANFs relative to the surrounding anatomy and the CI. Localization is challenging because the ANFs are so small that they are not directly visible in clinical imaging. We hypothesize that the position of the ANFs can be accurately inferred from the location of the internal auditory canal (IAC), which has high contrast in CT because the ANFs pass through this canal between the cochlea and the brain.

**Approach:**

Inspired by VoxelMorph, we propose a deep atlas-based IAC segmentation network. We create a single atlas in which the IAC and ANFs are pre-localized. Our network is trained to produce deformation fields (DFs) mapping coordinates from the atlas to new target volumes and that accurately segment the IAC. We hypothesize that DFs that accurately segment the IAC in target images will also facilitate accurate atlas-based localization of the ANFs. As opposed to VoxelMorph, which aims to produce DFs that accurately register the entire volume, our contribution is an entirely self-supervised training scheme that aims to produce DFs that accurately segment the target structure. This self-supervision is facilitated using a loss function inspired by the Mumford–Shah functional. We call our method Deep Atlas-Based Segmentation using Mumford-Shah (DABS-MS).

**Results:**

Results show that DABS-MS outperforms VoxelMorph for IAC segmentation. Tests with publicly available datasets for trachea and kidney segmentation also show significant improvement in segmentation accuracy, demonstrating the generalizability of the method.

**Conclusions:**

Our proposed DABS-MS method can accurately segment the IAC, which can then facilitate the localization of the ANFs. This patient-specific modeling of CI stimulation of the ANFs can help audiologists improve the CI programming, leading to better outcomes for patients with severe-to-profound hearing loss.

## 1 Introduction

Cochlear implants (CIs) are neural prosthetics used to treat patients with severe-to-profound hearing loss. The CI uses an array of 12 to 22 electrodes implanted into the patient’s cochlea to stimulate the auditory nerve fibers (ANFs) to induce the sensation of hearing. An external processor is worn by the patient, which processes sounds detected by a microphone and sends stimuli to the electrodes. After implantation, an audiologist optimizes the CI programming for a specific patient by adjusting various parameters. Typically, this process is entirely based on patient feedback and can require dozens of programming sessions, taking months or years, and often does not lead to optimal results.

We are now developing comprehensive patient-specific ANF stimulation models,^1^^,^^2^ which can assist audiologists with optimizing CI programming. These models are constructed by estimating the electric potentials across the ANFs induced by the CI electrodes, which allows for explicit modeling of the electro-neural interface (ENI) and the ability to assess neural health.^1^ However, this process requires accurate localization of the ANFs relative to surrounding anatomy and the CI. Localizing the ANFs is challenging because they are so small that they are not directly visible in clinical imaging.

In this work, we hypothesize that the position of the ANFs can be accurately inferred from the location of the internal auditory canal (IAC), which has high contrast in CT, because the ANFs pass through this canal between the cochlea and the brain. Inspired by VoxelMorph, we propose a deep atlas-based IAC segmentation network. We create a single atlas in which the IAC and ANFs are pre-localized. Our network is trained to produce deformation fields (DFs) mapping coordinates from the atlas to new target volumes, which accurately segment the IAC. We hypothesize that DFs that accurately segment the IAC in target images will also facilitate accurate atlas-based localization of the ANFs. As opposed to VoxelMorph, which aims to produce DFs that accurately register the entire volume, our novel contribution is an entirely self-supervised training scheme that aims to produce DFs that accurately segment the target structure. This self-supervision is facilitated using a Mumford–Shah functional-inspired loss function. We call our method Deep Atlas-Based Segmentation using Mumford-Shah (DABS-MS). In the remainder of this section, we present a discussion of related work.

### 1.1 Deep Atlas-Based Segmentation

Atlas-based image segmentation is a process in which an “atlas” image, which has pre-defined ground truth annotations of target anatomy, is automatically registered to a novel target image. Through this registration transformation, annotations on the atlas can be transferred to the given target image. The accuracy of the resulting annotations is directly related to the accuracy of the registration transformation. Atlas-based segmentation is especially common in medical image analysis, due to the similar relative positioning of many anatomical structures in humans.

Neural network architectures for image deformation are largely based on architectures for semantic image segmentation. The fully convolutional network, created by Long et al., is the basis for the most popular image segmentation network architectures.^3^ This architecture uses a series of down-sampling encoders and up-sampling decoders to classify each pixel in the image, maintaining the original resolution. The encoders produce feature channels that feed both into the encoder below it and the decoder at its same resolution level. In this way, the network is able to produce high-quality segmentations.

Segmentation neural networks are usually trained with images as inputs and a loss function that compares outputs to corresponding accurate ground truth labels. These networks require large amounts of annotated data to perform well. In Long et al.,^3^ the dataset sizes range from ∼1000 to 2500 images. Krizhevsky et al.,^4^ a breakthrough paper on deep learning in 2012, trained an impressively accurate neural network using 1.2 million images.

Unfortunately, accurately annotated datasets of this size are very difficult to obtain. Medical images, especially, are more difficult to capture and annotate. Ronneberger et al.^5^ proposed a modification to the fully convolutional neural network to help reduce the number of training images needed. In this new architecture, dubbed U-Net, the number of feature channels produced by encoders is increased greatly. This modification, combined with data augmentation, allowed the U-Net to provide very good results with only 30 to 35 training images.

Although Ronneberger et al.’s initial network was for 2D images, a 3D version was published by Çiçek et al.^6^ The U-Net architecture has become highly popular for segmenting 3D images.^7^^–^^9^

Ideally, neural networks are trained on a sizeable dataset with accurate ground truth annotations. Sometimes a sizeable set of images is available without strong annotations. In these cases, weak- or self-supervision learning methods have been proposed. Papandreou et al.^10^ proposed an expectation-maximization method for weakly supervised training. This method uses weak annotations, such as bounding boxes or image labels, and combines the use of weakly and strongly labeled images to train the network. Self-supervision strategies utilize loss function terms designed to evaluate the quality of network predictions to learn with unlabeled data.

U-Net-based architectures for semantic segmentation have been adapted for self-supervised atlas-based segmentation. A popular example is VoxelMorph, a framework proposed by Balakrishnan et al.^11^ The VoxelMorph architecture is based on the popular U-Net architecture^5^ but includes a modified final convolutional layer to produce a three-channel DF rather than a single-channel binary mask. VoxelMorph was proposed for both pair-wise registration and atlas-based segmentation. Although VoxelMorph was developed for brain MRI, subsequent papers have proposed using this framework for other modalities and anatomical objects of interest. For example, Miyake et al.^12^ used VoxelMorph to analyze the heart and lungs in Thoracic MDCT images.

In the current work, we propose a novel extension of VoxelMorph for atlas-based segmentation. The primary contribution is a custom loss function that facilitates more effective self-supervision learning for atlas-based segmentation of the particular class of structures that, when segmented accurately, are reasonably well represented locally by a Mumford–Shah energy functional.

### 1.2 Related Work

VoxelMorph for atlas-based segmentation is designed for self-supervised training on datasets with only an atlas ground truth. Xu et al. proposed the DeepAtlas framework, which provides a dynamic training strategy to be used on datasets with some amount of ground truth.^13^ They even report improvement over VoxelMorph when used on datasets that only have atlas ground truth labels. This mode is of primary interest to us because our goal is to create minimal ground truth for our datasets. When N=1, the DeepAtlas framework starts by training a registration-only network for atlas-based segmentation. Once converged, these atlas-based segmentations are used as ground truth segmentations for training a lightweight segmentation U-Net. Once converted, these two networks enter a joint training loop. In this loop, the segmentation network produces atlas and target segmentations. The deformation network produces a DF from the target image to the atlas image, which is used to deform both the target image and the predicted target segmentation. The loss function for the segmentation network minimizes the difference between the atlas ground truth segmentation and the predicted atlas segmentation, as well as the difference between the atlas ground truth segmentation and the deformed, predicted target segmentation. The loss function for the deformation network minimizes the difference between the atlas image and the deformed target image, the difference between the atlas segmentation and the deformed patient segmentation, and the gradient of the DF. Although this method reportedly outperforms VoxelMorph when using a single ground truth, it is unclear if it is by a statistically significant amount. In addition, during initial development, we found the training of DeepAtlas to take up to 4.5 times as long as VoxelMorph. The original paper also describes GPU memory issues that complicate and lengthen training times on datasets with bigger images. For these reasons, we did not choose DeepAtlas in this paper, opting for the more lightweight option of VoxelMorph instead. More details of our preliminary studies using DeepAtlas can be found in Appendix A.

Although CNNs and U-Nets have been the foundations for several computer vision tasks, newer works have introduced transformers into their image processing frameworks.^14^ Originally used in natural language processing (NLP) networks, transformers encode intensity and position information and have been shown to outperform CNNs in some studies. Dosovitskiy et al.^14^ introduced the vision transformer (ViT), which completely replaced CNNs for transformers in an image classification network. Although their results were comparable or better than state-of-the-art image recognition methods at the time on datasets with 14 million to 300 million images, they reported that ViT did not outperform ResNets on mid-sized datasets. Chen et al.^15^ introduced the TransUNet architecture, which replicated a standard U-Net but added transformer layers between the encoding and decoding phases. This method outperformed ViT for image segmentation when evaluated on a dataset of 3779 images, which demonstrated the effectiveness of transformers on mid-sized datasets. Swin-Unet introduced an image segmentation network that replicated the network architecture of the U-Net but replaced the convolution layers with Swin transformer layers.^16^^,^^17^ Swin-Unet outperformed TransUNet and U-Net for image segmentation on mid-sized datasets. Although these results are impressive, they are direct segmentation networks and do not perform our desired task of atlas-based segmentation. Swin-VoxelMorph is a framework proposed by Zhu et al., who used a Swin-Unet style network to output a DF instead of a direct segmentation.^18^ This method was shown to outperform VoxelMorph on a dataset of 1961 MRI scans. Although transformers have outperformed CNNs, U-Nets, and VoxelMorph in some studies, these studies use relatively large datasets. Section 2.1 details the exact metrics of our datasets, but their sizes range from 606 to only 40. Initial testing found Swin-VoxelMorph to perform comparably to a simple affine transform on our smallest dataset. Thus, in this work, we chose to use the traditional VoxelMorph architecture. More details of our preliminary studies using Swin-VoxelMorph can be found in Appendix A.

### 1.3 Contributions

The loss function originally proposed for self-supervised training of the VoxelMorph architecture consists of two terms—one to reward the similarity between the deformed and target image and another to reward the smoothness of the predicted DF.^11^

Both mean squared error (MSE) and cross correlation (CC) are common image similarity metrics. MSE measures the mean-squared differences in intensity for every pixel in the images. MSE is minimized when the intensities between two registered images are identical. By contrast, cross correlation is maximized when there exists a linear intensity relationship between two registered images.

The magnitude of the gradient of the DF has been proposed to measure its smoothness, and a loss function was proposed to minimize the mean magnitude of the gradient across the DF domain. This rewards the network for predicting smoothly varying DFs.^11^

Some papers have been published that add to, or modify, these loss terms. Zhu et al.^19^ proposed modified loss functions for the VoxelMorph framework, where the smoothing term is based on the Laplacian of the DF rather than the magnitude of the gradient of the DF.

Herein, we propose a new self-supervision term for atlas-based segmentation training that is inspired by the energy functional proposed by Chan and Vese based on the Mumford–Shah functional.^20^^,^^21^ A similarly inspired loss function was proposed by Kim and Ye as well as our group, for deep learning segmentation networks;^9^^,^^22^ however, to the best of our knowledge, this approach has not been applied to networks trained for atlas-based segmentation. In the remainder of the paper, we present our method and experiments designed to show its impact when applied to atlas-based segmentation of our IAC dataset as well as several other anatomical structures for which evaluation datasets were publicly available.

## 2 Methods

### 2.1 Datasets

We used three datasets for this paper: (1) an internal auditory canal (IAC) dataset; (2) the segmentation of thoracic organs at risk in the CT images dataset (SegTHOR) dataset;^23^ and (3) the 2021 kidney and kidney tumor segmentation challenge (KiTS21).^24^ Each dataset is comprised of 3D CT images. The appearance of these structures well matches the Mumford–Shah functional we propose as the region interior to the structures is relatively homogeneous in intensity compared with background structures. Thus, an accurate segmentation should overlap with an image region with minimized intensity variance.

#### 2.1.1 IAC

This dataset contains an atlas CT image, with a ground truth localization. It contains 606 patient CT images, where affine registrations to the atlas have been computed using a mutual information-based method^25^ as reported in previous works.^26^^,^^27^ The average voxel size of the dataset is 0.286  mm×0.286  mm×0.352  mm, with a variance of 0.009, 0.009, and $0.026\;{\mathrm{mm}}^{2}$ in each respective direction. The IAC dataset was split into 573 images for training, 16 for validation, and 17 for testing. Ground truth binary segmentations of the IAC were created for the 33 patients in the validation and test sets.

#### 2.1.2 SegTHOR

The SegTHOR dataset is part of the SegTHOR 2019 Challenge. This dataset provides 40 images with accurate ground truth segmentations for the trachea. The average voxel size of the dataset is 1.001  mm×1.001  mm×2.350  mm, with a variance of $0.009{\;\mathrm{mm}}^{2}$, $0.009{\;\mathrm{mm}}^{2}$, and $0.053{\;\mathrm{mm}}^{2}$ in each respective direction. One image was set aside as an atlas. The remaining 39 images were affinely registered to it using a mutual information-based approach.^25^ Due to the small size of this dataset, data augmentation was performed. Five images from the original dataset were set aside to serve as a test set and were not included in the data augmentation. The remaining images were augmented by applying a randomly generated DF. This process was done three times for each image. The resulting dataset was split into 109 images for training and 27 images for validation.

#### 2.1.3 Healthy KiTS21

Our final dataset comes from the 2021 Kidney and Kidney Tumor Segmentation Challenge. We chose not to focus on tumor segmentation at this time. Tumors are highly variable in shape, size, and position, making them unsuitable for localization with an atlas-based method. However, we selected 130 images of healthy kidneys to use to evaluate our method for atlas-based kidney segmentation. The average voxel size of these images is 3.316  mm×0.781  mm×0.781  mm, with a variance of 3.276, 0.011, and $0.011\;{\mathrm{mm}}^{2}$ in each respective direction. One image was chosen as the atlas, and the remaining 129 images were affinely registered to it using a mutual information-based approach.^25^ The Healthy KiTS21 dataset was split into 105 images for training, 12 images for validation, and 12 images for testing.

#### 2.1.4 Data Preprocessing

For all datasets, nonatlas images were affinely normalized with the atlas and then cropped to a 64 × 64 × 64 resolution cube around the centroid of the atlas segmentation. Voxel sizes were chosen to ensure the full object was visible in all images, resulting in 0.30 mm per voxel for the IAC dataset and 2.84375 mm per voxel for the others. Some pixels in the atlas-aligned images were outside the boundaries of the source images—in these cases, these pixels were assigned the mean value of the existing pixels.

In the VoxelMorph paper, it is recommended that the intensity values of the input data be normalized between 0 and 1. CT image intensity values can range from −1024 to 3071 Hounsfield Units (HU), and the intensity values of various tissue types are generally consistent across images. The dense bone of the cochlear region can reach up to 2000 HU, but metal artifacts can exceed 3000 HU.^28^ This high variance in maximum intensity values for this methodology suggests that normalizing each image based on its own minimum and maximum values will eradicate the important correspondence between tissues and intensity values found in CT images. To better standardize the datasets, a dataset-specific normalization was performed. For each dataset, constants $i_{\min}$ and $i_{\max}$ were heuristically chosen as the ideal minimum and maximum intensities across the training dataset. Then, as a preprocessing step, each training image T is normalized such that $T=(T-i_{\min})/(i_{\max}-i_{\min})$. Using ideal minimum and maximum values instead of the minimum and maximum values of individual images, the intensity values of the various tissue types will remain largely consistent across all images. In addition, most of the intensity values in the resulting images fall between 0 and 1 as is recommended in the VoxelMorph paper. Outlier intensities still exist outside that range.

### 2.2 Network Architecture

**Fig. 1:**
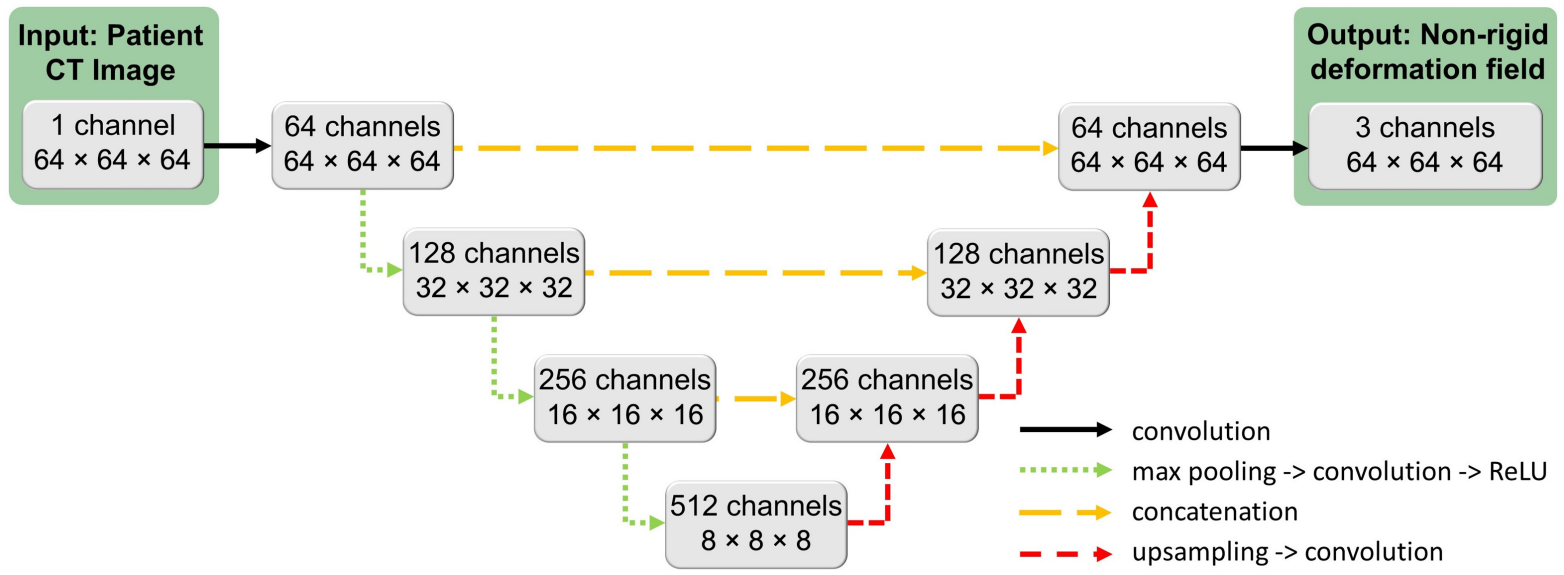
Network diagram.

We implemented the U-Net architecture adopted for VoxelMorph and inspired by Ronneberger et al. and Wolny et al..^5^^,^^7^^,^^11^ The architecture diagram can be seen in Fig. 1. The input to our network is a single-channel CT image T. The output is a nonrigid DF ϕ, which maps coordinates from the atlas space to the target image space. There are four levels of resolution, with three encoder levels and three decoder levels. There is a convolutional layer at the beginning of the network to extract feature channels from the input image. The output of the final convolutional layer has been modified from the single-channel probability map proposed with the original U-Net to instead output a three-channel DF.

### 2.3 Loss Functions

Similar to VoxelMorph, we adopt a cross-correlation (CC)-based image similarity metric.^11^ The equation for this term can be seen in Eq. (1), where μ denotes the mean

$$loss_{cc}=0.5-\frac{\left(\phi^{-1}(T)-\mu_{\phi^{-1}(T)}{)}^{T}(A-\mu_{A}\right)}{2\|\phi^{-1}(T)-\mu_{\phi^{-1}(T)}\|\|A-\mu_{A}\|}.$$ (1)

For improved regularization, we propose a modification to the smoothness term used with VoxelMorph.^11^ The smoothness term used with the VoxelMorph framework is simply the mean-squared magnitude of the gradient of the DF, which can be seen in Eq. (2)

$$loss_{grad}=\frac{1}{3N}.\overset{N}{\underset{i}{\sum }}\|\nabla \phi_{i}{\|}^{2}.$$ (2)

However, it assigns equal weight to all areas of the image domain. For our purpose, atlas-based segmentation of a single structure, we propose modifying this term to prioritize smoothness near the target structure.

To enable measuring proximity to the target, we compute a signed Euclidean distance map D for the atlas segmentation $S_{A}$. A weighting term leveraging this distance map is defined in Eq. (3)

$$W=0.5+\frac{t_{U}-\max(t_{L},\min(t_{U},|D|))}{2(t_{U}-t_{L})}.$$ (3)

As shown in the equation, the weight is a function of the magnitude of the distance map, with values clamped between a lower threshold $t_{L}$ and an upper threshold $t_{U}$. We chose these values to be 1 and 4 mm for our datasets. This result is used as a weighting for the deformation smoothness term that more heavily weights smoothness around the border of the atlas segmentation, as shown in the loss term in Eq. (4).

$$loss_{wgrad}=\frac{1}{3N}\overset{N}{\underset{i}{\sum }}\|W_{i}\nabla \phi_{i}{\|}^{2}\;.$$ (4)

In addition, inspired by Kim and Ye, we propose a new Mumford–Shah functional-based loss term (MS).^22^ This term assumes that the interior of the desired segmentation has a distinguishable average image intensity compared with the average image intensity of the exterior region. Equations (5)–(9) define this term. It uses the atlas segmentation mask A to delineate the foreground of the atlas-based segmentation on the deformed target image $\phi^{-1}(T)$. The final loss term in Eq. (9) aims to minimize the intra-class variance of the intensity distributions of the image regions labelled as belonging to foreground and background by the atlas-based segmentation

$$\mu_{int}=\frac{\sum_{i=1}^{N}\phi^{-1}(T{)}_{i}*A_{i}}{\sum_{i=1}^{N}A_{i}},$$ (5)

$$\mu_{ext}=\frac{\sum_{i=1}^{N}\phi^{-1}(T{)}_{i}*(1-A_{i})}{\sum_{i=1}^{N}(1-A_{i})},$$ (6)

$$\sigma_{int}^{2}=\frac{\sum_{i=1}^{N}(\phi^{-1}(T{)}_{i}-\mu_{int}{)}^{2}*A_{i}}{\sum_{i=1}^{N}A_{i}},$$ (7)

$$\sigma_{ext}^{2}=\frac{\sum_{i=1}^{N}(\phi^{-1}(T{)}_{i}-\mu_{ext}{)}^{2}*(1-A_{i})}{\sum_{i=1}^{N}(1-A_{i})},$$ (8)

$$loss_{MS}=\sigma_{int}^{2}+\sigma_{ext}^{2}.$$ (9)

#### 2.3.1 Combined loss functions

The loss function used in the VoxelMorph framework can be seen in Eq. (10). Our proposed loss function can be seen in Eq. (11)

$$loss_{VXM}=\omega_{cc}*loss_{cc}+\omega_{grad}*loss_{grad},$$ (10)

$$loss_{new}=\omega_{cc}*loss_{cc}+\omega_{wgrad}*loss_{wgrad}+\omega_{MS}*loss_{MS}.$$ (11)

A greedy-type optimization strategy with a fixed set of hyperparameter values was performed to fine-tune the loss function weights. The cross-correlation weight was kept constant at 1.0, and the weight of the gradient term was tested at 0.1, 0.5, 1.0, 2.0, and 10.0, without the Mumford–Shah (MS) term. This sweep was also performed for the weighted gradient. The best terms from this sweep were then held constant, and the MS term was added. A sweep was performed for the weight of the MS term along the same range of weights. The best weights can be seen in Table 1.

**Table 1:** Each row shows the best weights for each combination of terms for each dataset. A blank cell indicates that the corresponding loss term was not included in that combination.

Dataset	$\omega_{cc}$	$\omega_{grad}$	$\omega_{wgrad}$	$\omega_{MS}$
IAC	1.0	0.5	—	—
IAC	1.0	0.5	—	0.5
IAC	1.0	—	0.5	—
IAC	1.0	—	0.5	0.5
SegTHOR	1.0	1.0	—	—
SegTHOR	1.0	1.0	—	0.5
SegTHOR	1.0	—	1.0	—
SegTHOR	1.0	—	1.0	0.5
KiTS21	1.0	1.0	—	—
KiTS21	1.0	1.0	—	1.0
KiTS21	1.0	—	2.0	—
KiTS21	1.0	—	2.0	0.5

## 3 Evaluation Metrics

The output DF from the network can be used to project the atlas IAC segmentation surface onto the target volume. We compare this resulting segmentation to ground truth segmentations using the 95^th^ Percentile Hausdorff distance to quantify surface distance errors and Dice similarity coefficients to quantify volumetric overlap errors. The statistical significance of our results was evaluated with the dependent t-test for paired samples.

### 3.1 Special Considerations for the IAC

The IAC is a structure with no true anatomical boundary separating it centrally from the brain. Any such boundaries delineated in the ground truth segmentation are completely arbitrary. Comparing the arbitrary boundary of the network result to the arbitrary boundary of the ground truth would not be informative. Conversely, including this area in our metrics can occlude the true performance of our results in the areas that are actually important for our downstream task of ANF localization. Thus, for the IAC, the region not important for our ANF localization was hand-annotated in our validation and testing datasets, and this region is excluded when computing the evaluation metrics. In addition, a tissue-based segmentation was used for the Mumford–Shah term, where all of the soft tissue regions in the images were masked, rather than just the IAC. This was done because the boundaries between some of these regions are arbitrary. If the Mumford–Shah term were to encourage the network to line up the arbitrary boundaries with visible boundaries between bone and soft tissue, that would provide undesirable segmentations. For these reasons, a modified atlas segmentation was used for the MS term on the IAC dataset.

For the SegTHOR and Healthy KiTS21 datasets, the entire image is included in the evaluation metrics, and no such modification was made to the atlas segmentation for the MS term.

### 3.2 Training Strategy

The choice of initial weights of a convolutional neural network prior to training can have a significant impact on final network performance, and even repeat optimizations starting from identical initial weights can lead to variable results.^29^^–^^31^ Henderson et al.^32^ discussed how this lack of consistency can make it difficult to interpret results. To mitigate these concerns, in our experiments, pseudo-random number generators for all code libraries were seeded with a constant at the start of each trial. In addition, each training configuration was evaluated five times. In Sec. 4, we report median results across training runs to determine the best configuration.

## 4 Results

**Fig. 2:**
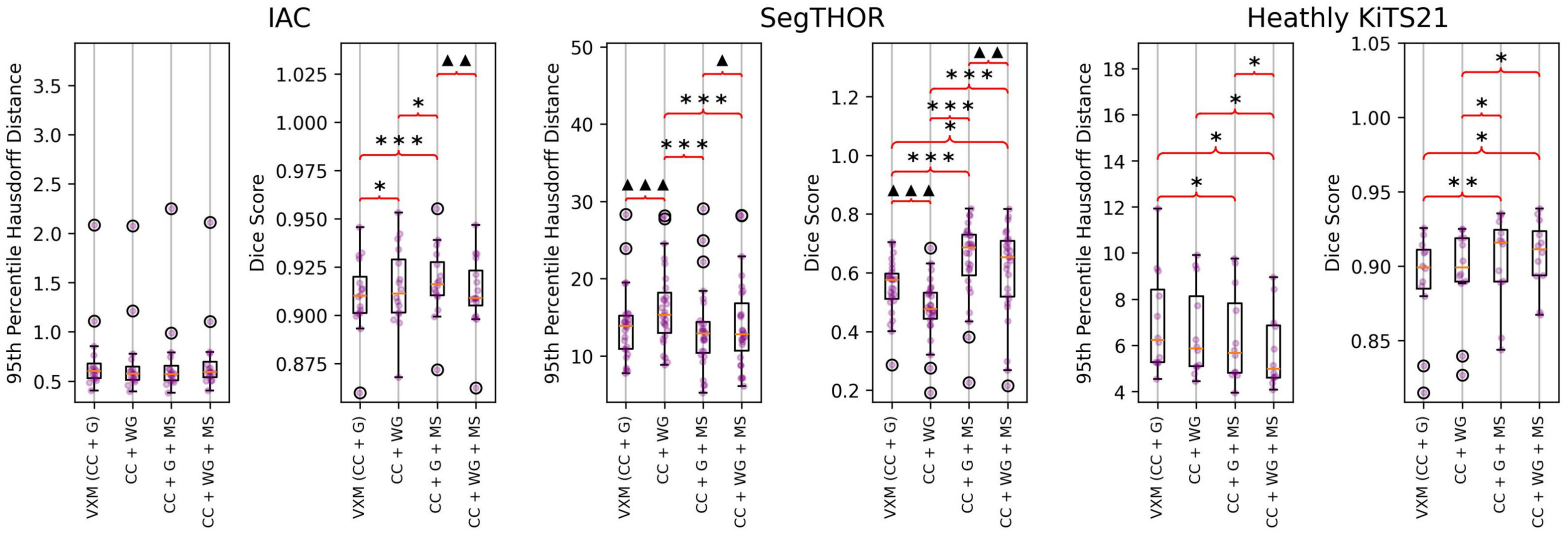
Ablation study results. Bracket indicates a significant difference. The asterisk symbol indicates that the right result is significantly better than the left. The triangle symbol indicates that the right is significantly worse. A single symbol indicates the t-test resulted in a p-value less than 0.05. Two symbols indicate a $p\text{-}\text{value}<1\times 10^{-3}$. Three indicate a $p\text{-}\text{value}<1\times 10^{-5}$.

After performing a hyperparameter search for the terms in our proposed loss function as described in Sec. 2.3.1, we performed an ablation study to evaluate the contribution of each of our proposed loss terms on the validation data for each dataset. Statistical significance of the difference in performance between trials was evaluated using paired t-tests. The results can be seen in Fig. 2.

According to the ablation study, the MS loss term provided significant improvement for all datasets. The weighted gradient term caused a significant decrease in performance in all term combinations for the SegTHOR dataset. Although the weighted gradient term improved performance when compared with the regular gradient for the IAC dataset, the weighted gradient used with the MS term created significantly worse results than the regular gradient with the MS term. The weighted gradient term insignificantly improved performance on the Healthy KiTS21 dataset on its own, but when combined with the MS term, it showed significant improvement.

For each dataset, the final “best” loss function after dataset-specific loss function customization can be seen in Eqs. (12)–(14).

$$loss_{IAC}=loss_{cc}+0.5loss_{grad}+0.5loss_{MS},$$ (12)

$$loss_{SegTHOR}=loss_{cc}+loss_{grad}+0.5loss_{MS},$$ (13)

$$loss_{HKiTS21}=loss_{cc}+2.0loss_{wgrad}+0.5loss_{MS}.$$ (14)

**Fig. 3:**
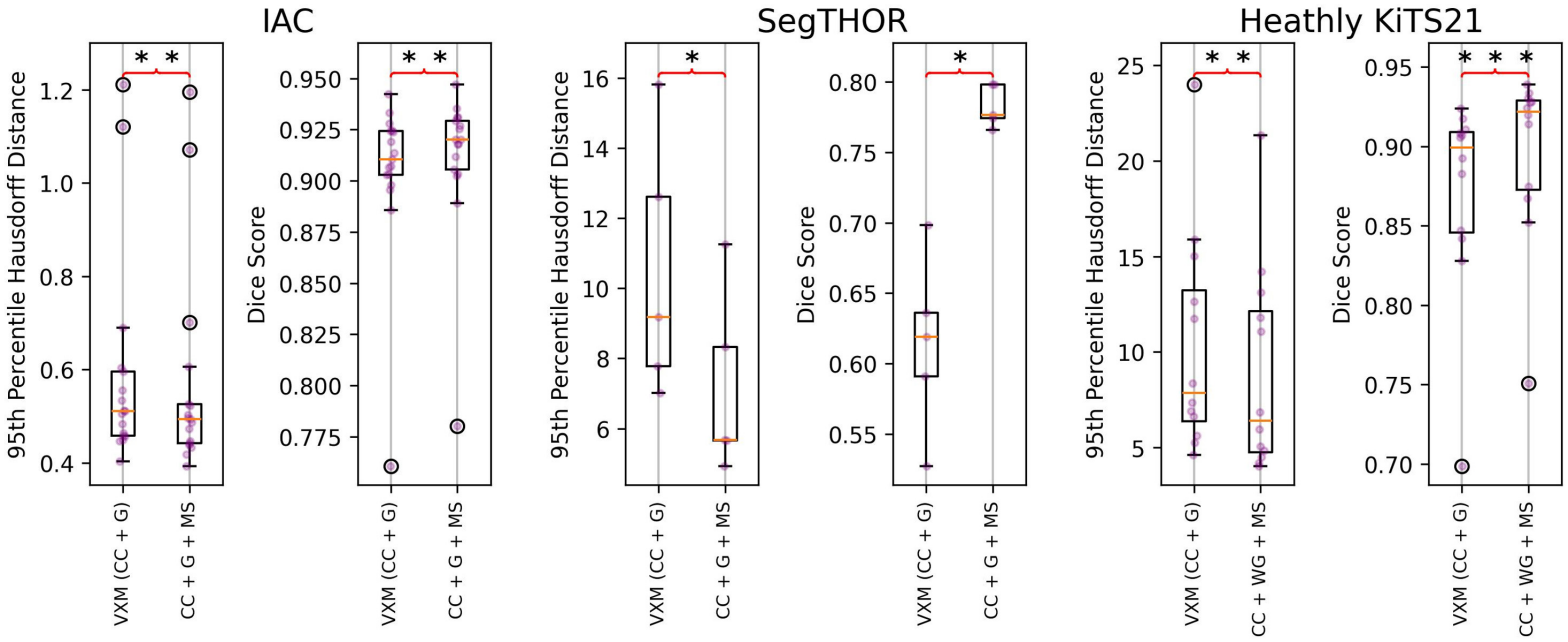
Results of our proposed method versus VoxelMorph (VXM). Symbols follow the same legend as the ablation study.

Figure 3 shows the results of a network trained with our final loss functions [see Eqs. (12)–(14)] on the testing datasets. These results are compared with those from a network trained using the loss function used in the VoxelMorph framework [see Eq. (10)]. The significance is displayed in the same manner as the ablation study in Fig. 2.

Table 2 shows median values for each metric evaluated on VoxelMorph and DABS-MS.

**Table 2:** Median (standard deviation) values for each metric on each dataset on each method. 95HD stands for 95th Percentile Hausdorff Distance.

		IAC	SegTHOR	KiTS21
Median 95HD ↓	VXM	0.511 (0.222)	9.177 (3.283)	7.863 (5.520)
	DABS-MS	**0.494 (0.220)**	**5.667 (2.349)**	**6.397 (5.191)**
Median Dice score ↑	VXM	0.910 (0.039)	0.619 (0.056)	0.899 (0.060)
	DABS-MS	**0.920 (0.036)**	**0.777 (0.013)**	**0.922 (0.052)**

The best result for each metric is bolded.

**Fig. 4:**
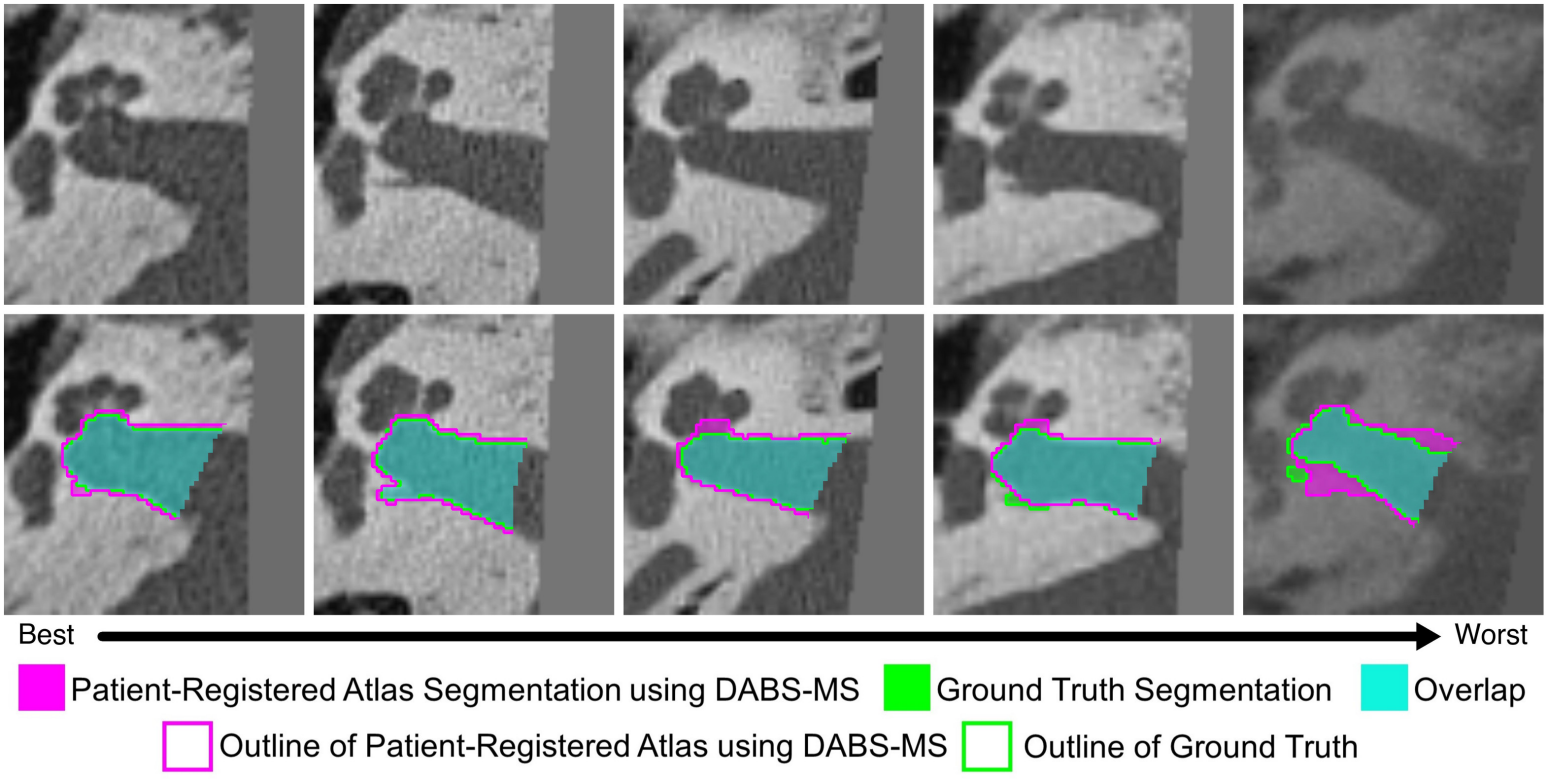
Axial slices of our proposed method on IAC testing data, on a selection of cases showing the full range of performance.

**Fig. 5:**
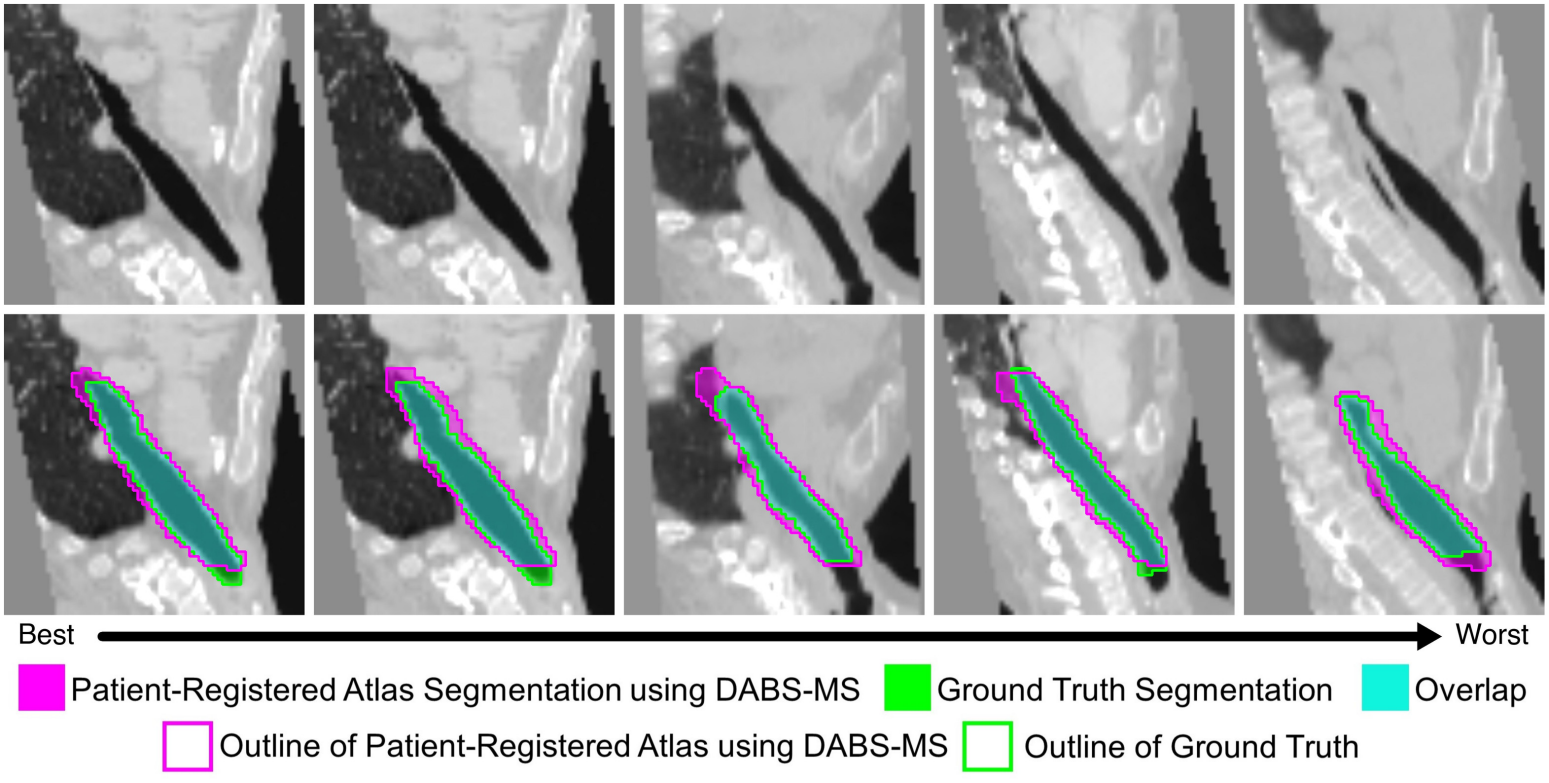
Coronal slices of our proposed method on SegTHOR testing data, on a selection of cases showing the full range of performance.

**Fig. 6:**
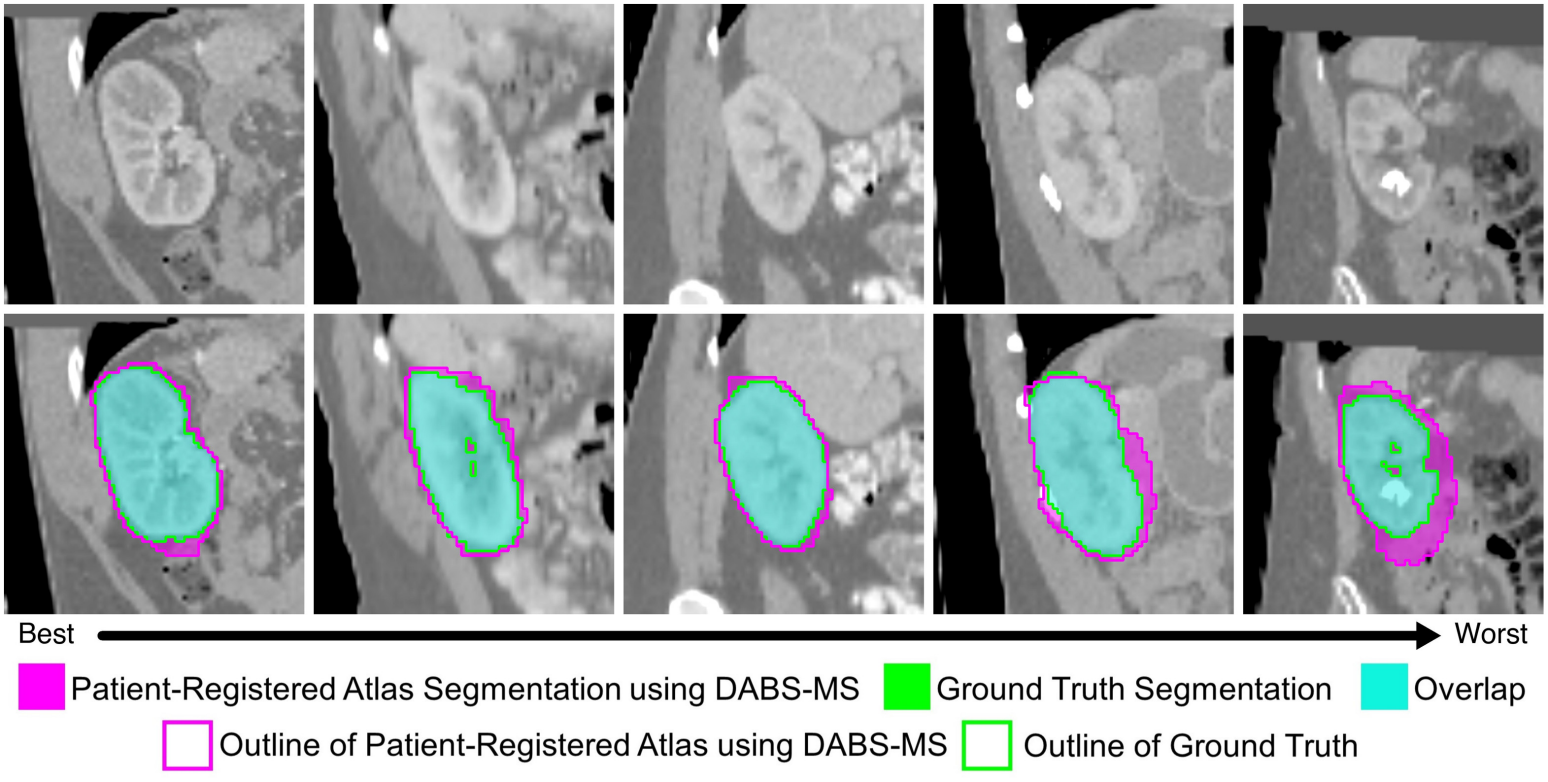
Axial slices of our proposed method on Healthy KiTS21 testing data, on a selection of cases showing the full range of performance.

Qualitative results for each dataset can be seen in Figs. 4–6. These are all 3D volumes, but 2D slices have been selected to highlight the biggest areas of the segmentations. Each figure shows results from five cases, selected uniformly across the range of Dice scores. The cases span from the best performance on the left to the worst performance on the right. The exact 95HD and Dice scores for these cases can be seen in Table 3. For the IAC dataset, errors are fairly uniformly distributed and relatively small. For the SegTHOR datasets, the largest errors typically occur at the superior boundary, where there is a lack of a true anatomical boundary to indicate the location of the boundary of the structure. For the KiTS21 dataset, large errors are observed in some cases likely due to the smaller volume of surrounding darker intensity tissue.

**Table 3:** 95HD and Dice scores for the cases shown in Figs. 4–6.

	Metric	Case 1 (best)	Case 2	Case 3	Case 4	Case 5 (worst)
IAC	95HD	0.3947	0.4769	1.1963	0.4338	1.1008
	Dice	0.9510	0.9299	0.9201	0.9043	0.7636
SegTHOR	95HD	5.7335	5.9053	5.7398	4.5746	8.9933
	Dice	0.8162	0.7979	0.7824	0.7661	0.7494
Healthy KiTS21	95HD	6.2096	5.8904	12.0868	11.4862	21.3502
	Dice	0.9453	0.9302	0.9206	0.8715	0.7331

## 5 Discussion and Conclusion

The results indicate the significant promise of our proposed extensions to the loss function used in the VoxelMorph framework. All testing accuracy metrics on all datasets show significant improvement relative to VoxelMorph.

Our approach has limitations in terms of the types of anatomical structures for which it is applicable. For structures that do not exhibit intensity homogeneity or do not have a distinct difference in average intensity values between foreground and background, our proposed loss function will not improve upon the more general loss function used in the VoxelMorph framework. However, we find that for atlas-based segmentation of structures that are reasonably well represented by a Mumford–Shah functional, our method provides significant improvement.

Future projects include utilizing our proposed method on the IAC dataset to localize ANFs. As ANFs are enclosed by the IAC, we hypothesize that an accurate IAC atlas-based segmentation should facilitate an accurate localization of the ANFs. We hope that the resulting ANF localizations will provide essential information for our patient-specific ANF activation models.

## 6 Appendix A: Preliminary Studies

Within this appendix, we examine other methods and options for architecture and briefly discuss why they were omitted from the main body of this paper.

### 6.1 DeepAtlas

DeepAtlas^13^ uses a deformation network and a segmentation network that are trained separately initially, then together. For the deformation network, we used VoxelMorph with the tuned weights described in Table 1. The image similarity metric, $L_{i}$, was the cross-correlation, and the regularization term, $L_{r}$, was the gradient term in Eq. (2). For the segmentation network, we used a 3D segmentation U-Net^7^ with the soft-multiclass Dice loss described in the DeepAtlas paper. The VoxelMorph network was trained to produce DFs, from which atlas-based segmentations were produced for all training data. Then, the segmentation network was trained for binary mask segmentation using these atlas-based segmentations are ground truths. Once both networks had converged, we began the process of training them simultaneously. This process introduces two new loss terms: anatomy loss $L_{a}$ and supervised loss $L_{sp}$, which both use the soft-multiclass Dice to compare their inputs. The anatomy loss compares the deformed predicted patient segmentation to the known atlas segmentation, promoting the accuracy of the DF and well as the predicted patient segmentation. The supervised loss compares the predicted atlas segmentation to the known atlas segmentation. When used with only a single ground truth, the loss function for the segmentation network is as follows, where $\lambda_{a}$ and $\lambda_{sp}$ are the weights for $L_{a}$ and $L_{sp}$, respectively, SegNet is the segmentation network, ϕ is the DF outputted by the DF, $S_{A}$ is the known atlas segmentation, $I_{A}$ is the atlas image, and $I_{P}$ is the patient image

$$seg_{loss}=\lambda_{a}L_{a}(\phi^{-1}(SegNet(I_{P})),S_{A})+\lambda_{sp}L_{sp}(SegNet(I_{A}),S_{A}).$$ (15)

The deformation loss is as follows, with $\lambda_{r}$ being the weight for $L_{r}$

$$def_{loss}=L_{i}(\phi^{-1}(I_{P}),I_{A})+\lambda_{r}L_{r}(\phi^{-1})+\lambda_{a}L_{a}(\phi^{-1}(SegNet(I_{P})),S_{A}).$$ (16)

**Fig. 7:**
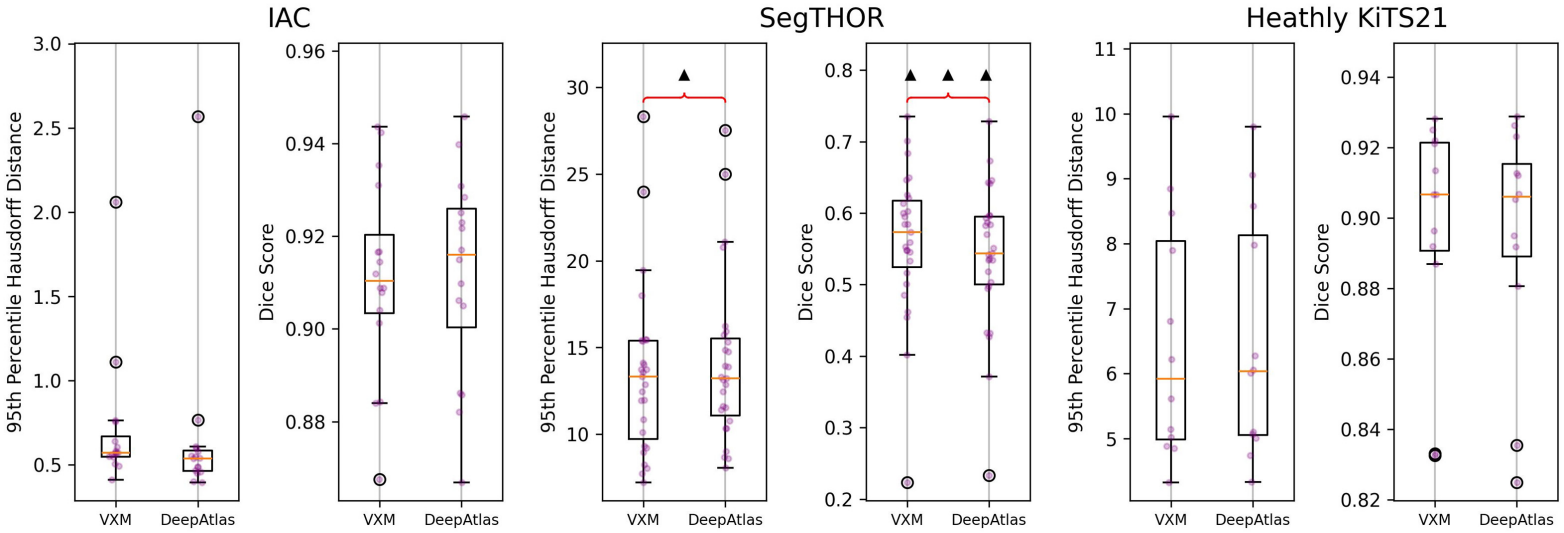
VoxelMorph versus DeepAtlas on validation data. No bracket indicates no significant difference. The asterisk symbol indicates the right result is significantly better than the left. The triangle symbol indicates that the right is significantly worse. A single symbol indicates the t-test resulted in a p-value less than 0.05. Two symbols indicate a $p\text{-}\text{value}<1\times 10^{-3}$. Three indicate a $p\text{-}\text{value}<1\times 10^{-5}$.

We used 1.0 for $\lambda_{a}$ and $\lambda_{sp}$ and used the optimal $\omega_{g}$ from Table 1 as $\lambda_{r}$ for each dataset for our initial run. Just this co-training step took up to 4.5 times as long as it took to train VoxelMorph. The results on validation data can be seen in Fig. 7.

**Fig. 8:**
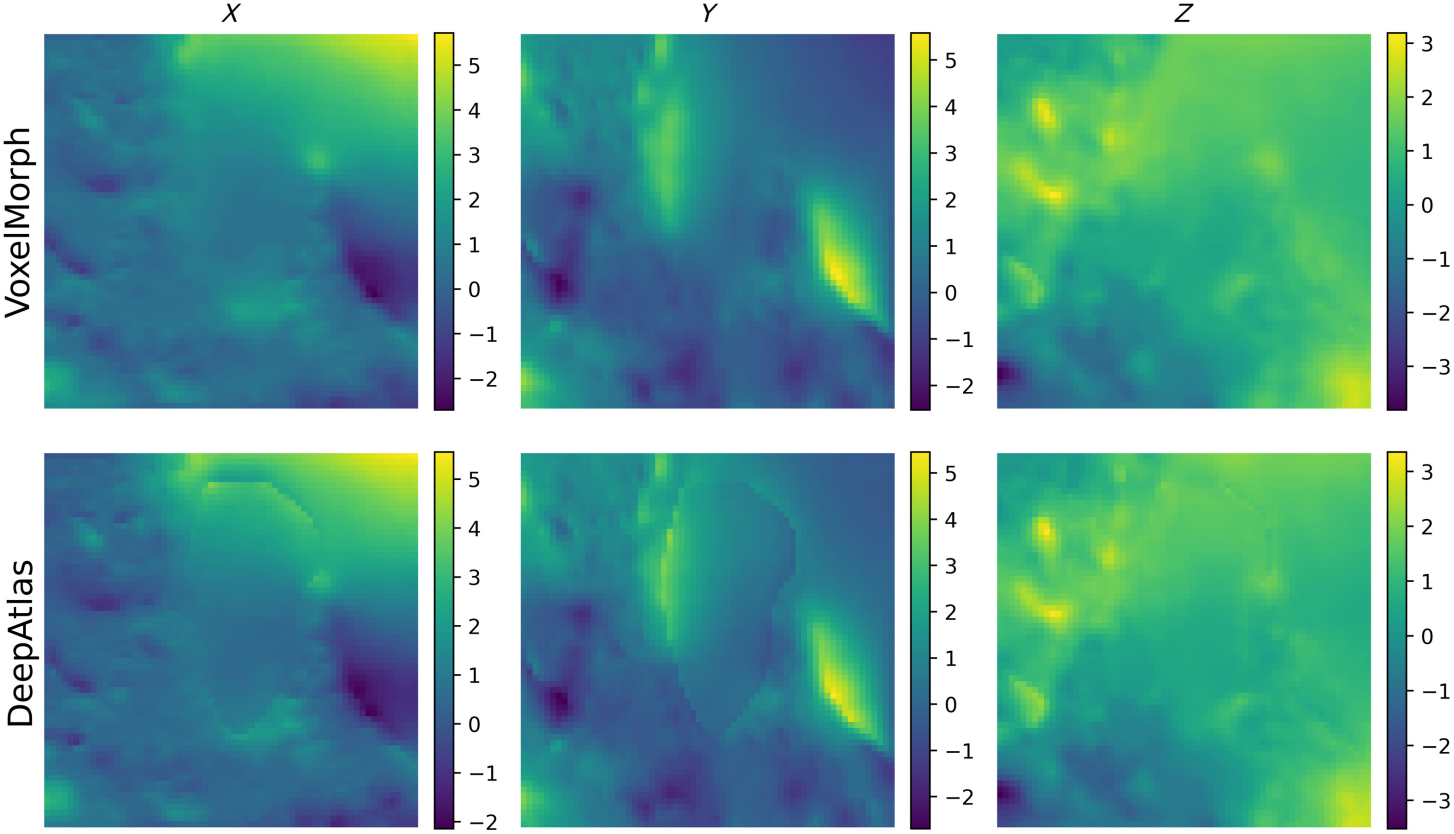
Comparison of a central axial slice of the DFs for one case from VoxelMorph and DeepAtlas.

DeepAtlas does not provide significant improvement for any of our datasets when evaluated with a dependent t-test, in fact, performing significantly worse on SegTHOR. In addition, DeepAtlas produced an artifact in the DF around the atlas segmentation, seen in Fig. 8. Even if this slightly increases segmentation accuracy for the IAC, this is contrary to our goal of making smooth, realistic DFs that can be used for other labels in the same area without additional training.

**Fig. 9:**
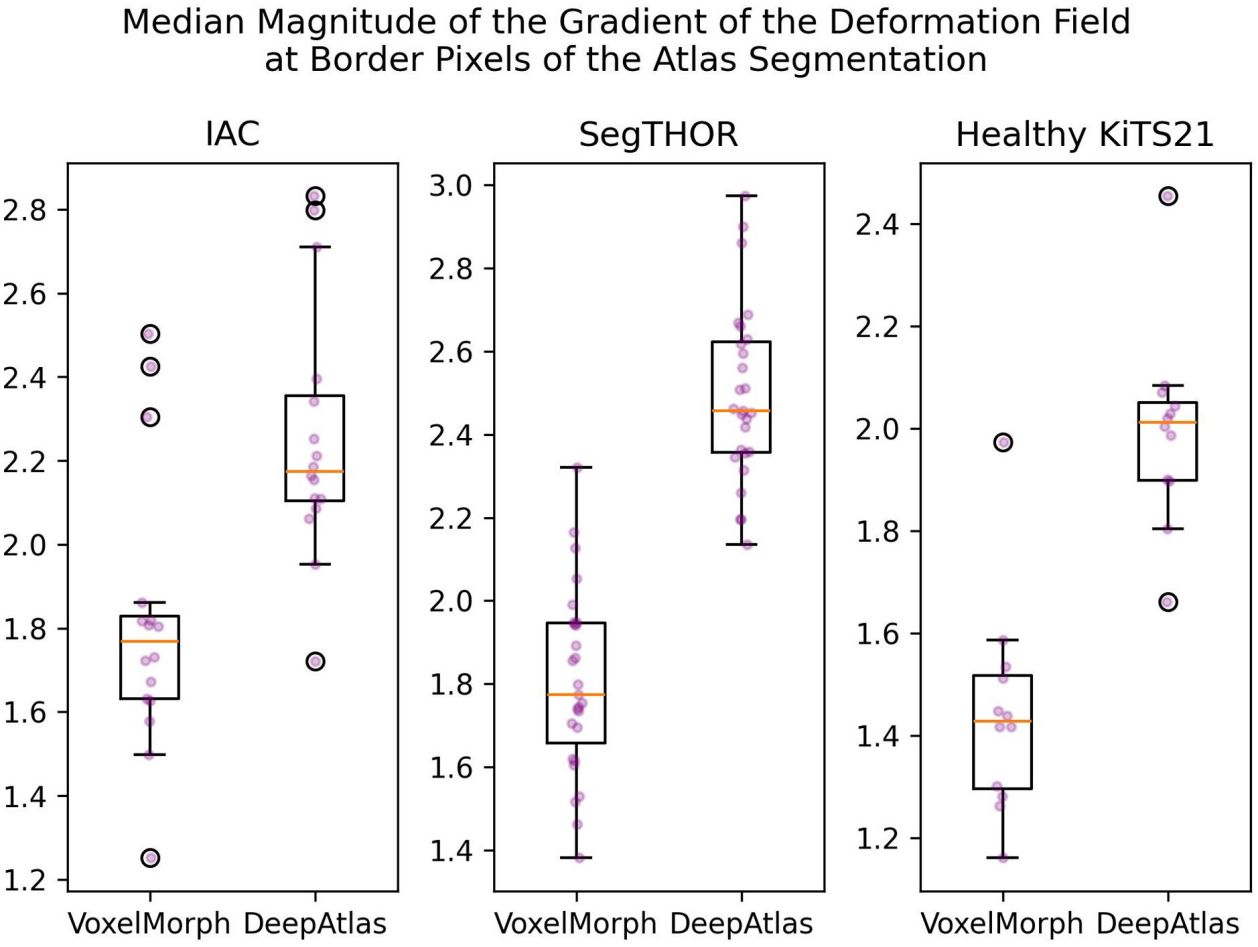
Analysis of the magnitude of the gradient of the DFs for VoxelMorph versus DeepAtlas.

This artifact can be seen for all three datasets. We calculated the magnitude of the gradient of the DF at the border pixels of the atlas segmentation and found that DeepAtlas trended toward a higher median gradient. This can indicate less realistic DFs. These data can be seen in Fig. 9.

Due to this concerning artifact and significant training time, we did not pursue this method further.

### 6.2 Transformers

**Fig. 10:**
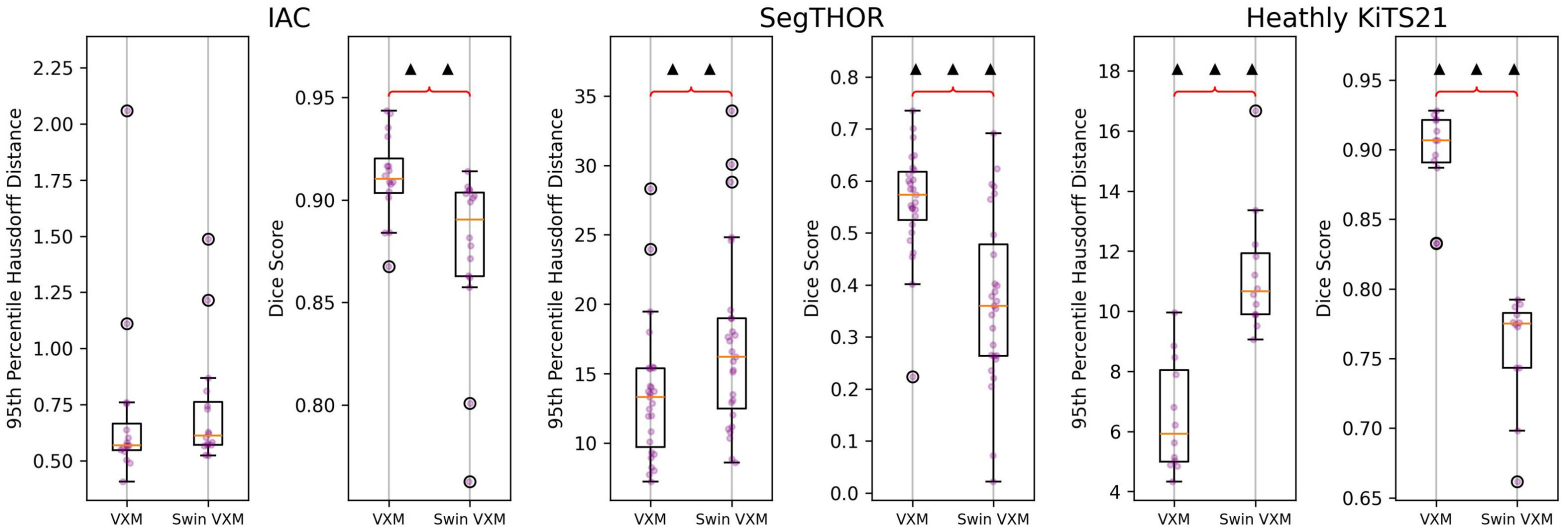
VoxelMorph (VXM) versus Swin-VoxelMorph (SVXM). No bracket indicates no significant difference. The asterisk symbol indicates that the right result is significantly better than the left. The triangle symbol indicates that the right is significantly worse. A single symbol indicates that the t-test resulted in a p-value<0.05. Two symbols indicate a $p\text{-}\mathrm{value}<1\times 10^{-3}$. Three indicate a $p\text{-}\text{value}<1\times 10^{-5}$.

As mentioned in Sec. 1, transformers have been successfully adapted from natural language processing tasks to several image processing applications. Though often used with much bigger datasets than our own, we briefly looked into Swin-VoxelMorph^18^ for our application. This takes the traditional U-Net structure of VoxelMorph and replaces the convolutional layers with Swin blocks. We trained this framework on all three datasets with Eq. (10) as the loss function. We performed a hyperparameter sweep and found the optimal $\omega_{g}$ to be 0.5, 1.0, and 0.05 for the IAC, SegTHOR, and Healthy KiTS21 datasets. The comparison of these results to VoxelMorph can be seen in Fig. 10.

**Fig. 11:**
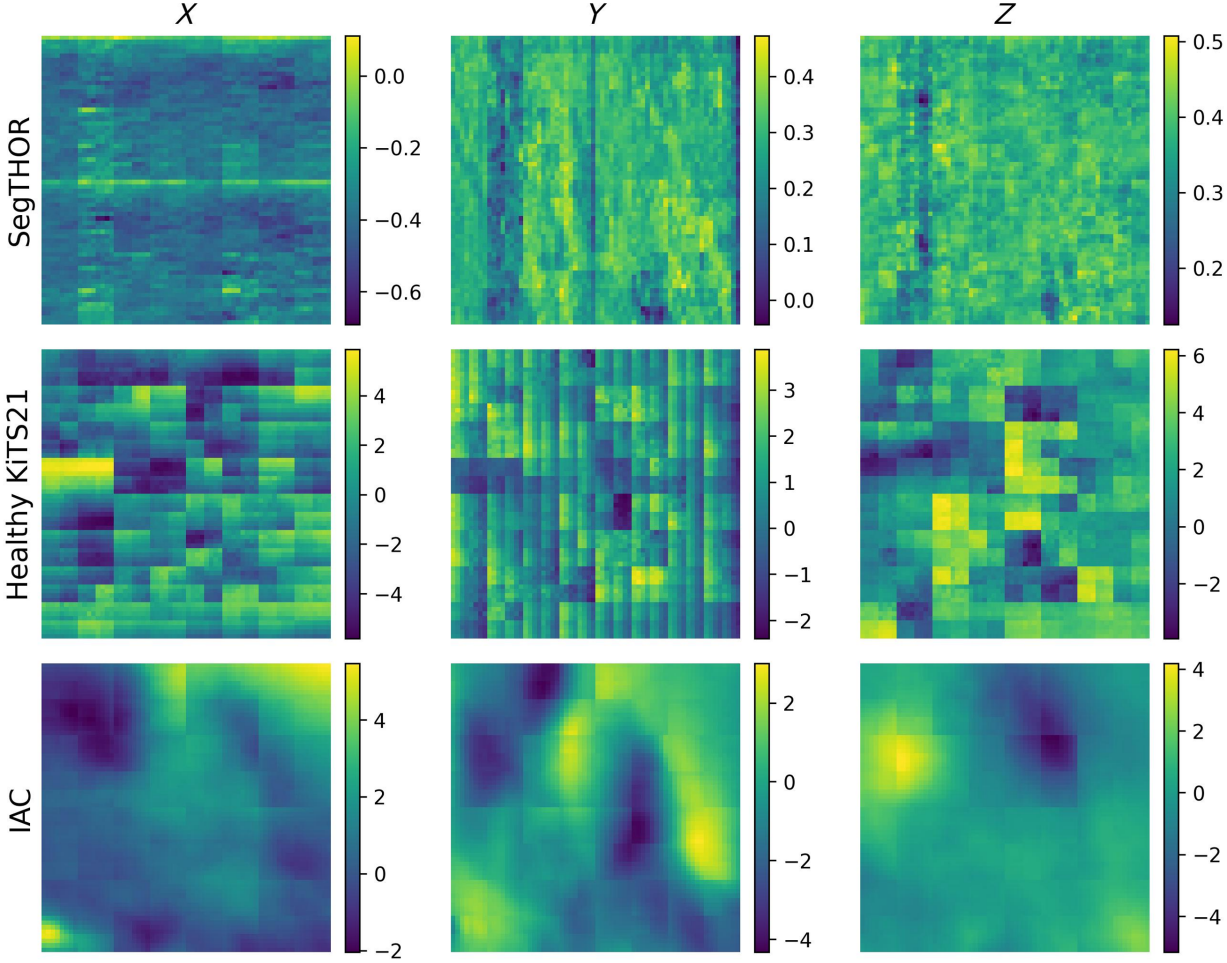
Comparison of a central axial slice of the Swin-VoxelMorph DFs for each dataset.

Overall, Swin-VoxelMorph performed significantly worse for our datasets than standard VoxelMorph. This is not unexpected due to the small size of our datasets. In addition, the resulting DFs from this method had sharp deformations around the edges of the patches, which can be seen in Fig. 11.

This artifact is less noticeable with the IAC dataset than the others, which suggests that more data will lessen this pattern. Unfortunately, we are limited to the available data. For these reasons, we did not pursue a transformed-based architecture for our method.

### 6.3 Traditional Methods

**Fig. 12:**
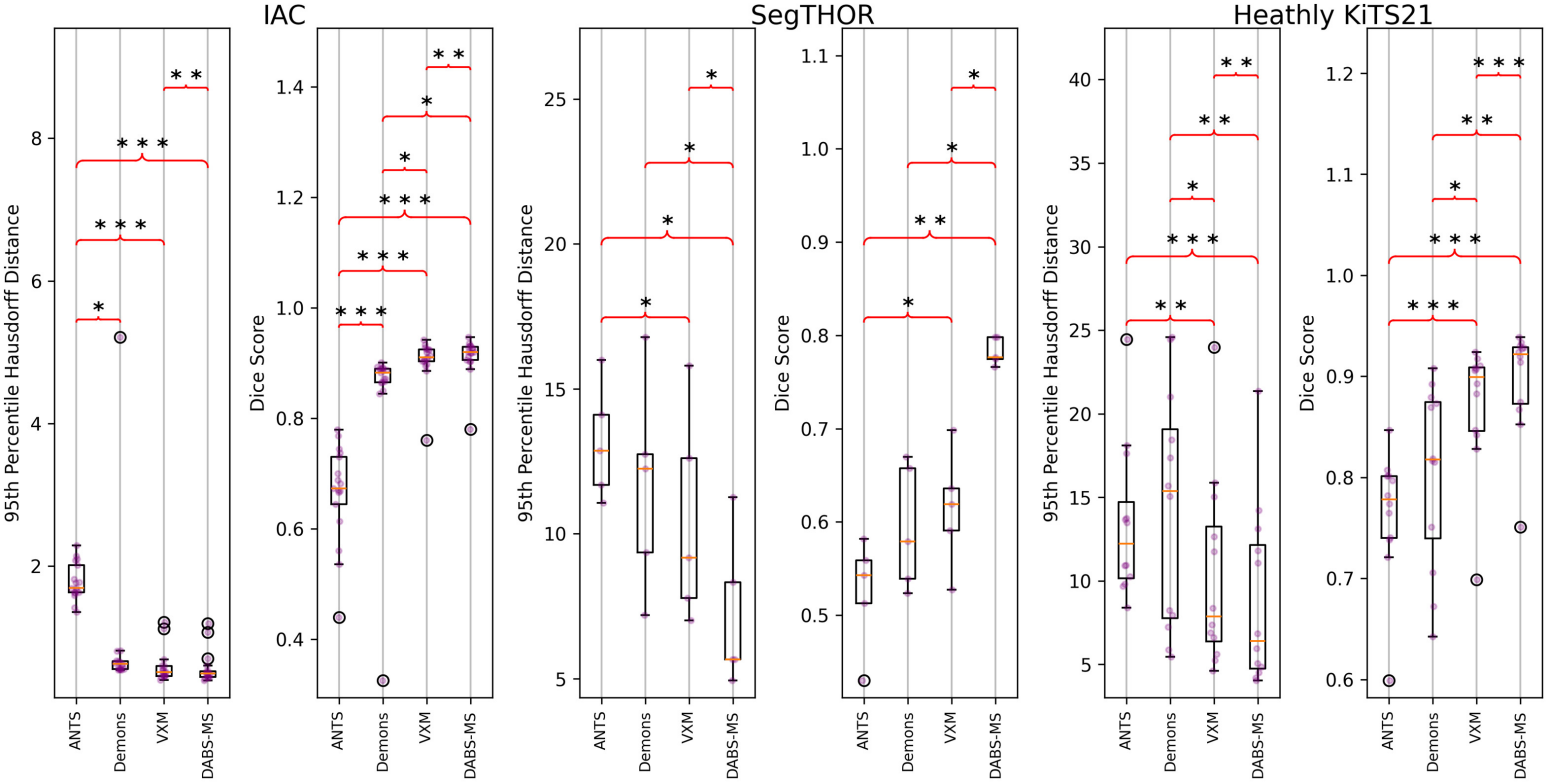
Comparison of traditional methods to neural network-based methods. No bracket indicates no significant difference. The asterisk symbol indicates the right result is significantly better than the left. The triangle symbol indicates that the right is significantly worse. A single symbol indicates the t-test resulted in a p-value<0.05. Two symbols indicate a $p\text{-}\text{value}<1\times 10^{-3}$. Three indicate a $p\text{-}\text{value}<1\times 10^{-5}$.

Traditional methods have been proposed for nonrigid image registration. VoxelMorph has been shown to be comparable or better than popular traditional methods, with much shorter runtimes.^11^ We tested our method and VoxelMorph against ANTs Syn^33^ and Demons,^34^ and the results on the validation data for our three datasets can be seen in Fig. 12.

We can see from initial tests that the neural-network-based methods outperform the traditional methods on our datasets. Although further tuning could be done to increase the performance of the traditional methods, these results show an initial comparison and the potential of deep learning methods.

## Data Availability

The code for this project can be found at github.com/hgmason/DABS-MS. Patient consent was not obtained to permit publicly sharing the IAC dataset. The SegTHOR^23^ dataset can be found here: https://competitions.codalab.org/competitions/21145. The KiTS21^24^ dataset can be found here: https://kits-challenge.org/kits21/. Note that only kidneys without tumors were used. These datasets were preprocessed for this task, as described in Sec. 2.1.4. Preprocessed versions of the datasets will be made available upon request.
